# The tanning hormone, bursicon, does not act directly on the epidermis to tan the *Drosophila* exoskeleton

**DOI:** 10.1186/s12915-020-0742-5

**Published:** 2020-02-19

**Authors:** Justin Flaven-Pouchon, Javier V. Alvarez, Candy Rojas, John Ewer

**Affiliations:** Instituto de Neurociencia, Universidad de Valparaíso, Valparaiso, Chile

**Keywords:** Cuticle, Melanization, Sclerotization, *rickets* gene, Neuropeptide

## Abstract

**Background:**

In insects, continuous growth requires the periodic replacement of the exoskeleton. Once the remains of the exoskeleton from the previous stage have been shed during ecdysis, the new one is rapidly sclerotized (hardened) and melanized (pigmented), a process collectively known as tanning. The rapid tanning that occurs after ecdysis is critical for insect survival, as it reduces desiccation, and gives the exoskeleton the rigidity needed to support the internal organs and to provide a solid anchor for the muscles. This rapid postecdysial tanning is triggered by the “tanning hormone”, bursicon. Since bursicon is released into the hemolymph, it has naturally been assumed that it would act on the epidermal cells to cause the tanning of the overlying exoskeleton.

**Results:**

Here we investigated the site of bursicon action in *Drosophila* by examining the consequences on tanning of disabling the bursicon receptor (encoded by the *rickets* gene) in different tissues. To our surprise, we found that rapid tanning does not require *rickets* function in the epidermis but requires it instead in peptidergic neurons of the ventral nervous system (VNS). Although we were unable to identify the signal that is transmitted from the VNS to the epidermis, we show that neurons that express the *Drosophila* insulin-like peptide ILP7, but not the ILP7 peptide itself, are involved. In addition, we found that some of the bursicon targets involved in melanization are different from those that cause sclerotization.

**Conclusions:**

Our findings show that bursicon does not act directly on the epidermis to cause the tanning of the overlying exoskeleton but instead requires an intermediary messenger produced by peptidergic neurons within the central nervous system. Thus, this work has uncovered an unexpected layer of control in a process that is critical for insect survival, which will significantly alter the direction of future research aimed at understanding how rapid postecdysial tanning occurs.

**Electronic supplementary material:**

The online version of this article (10.1186/s12915-020-0742-5) contains supplementary material, which is available to authorized users.

## Background

The insect exoskeleton (or cuticle) provides support for the animal’s organs and is also involved in a number of critical functions ranging from providing resistance to pathogens and desiccation to social communication and has likely contributed to this group’s evolutionary success. At the end of each molt, insects shed the remains of the old cuticle during ecdysis, then rapidly expand, pigment (melanize), and harden (sclerotize) the new exoskeleton. The molecular pathways that cause cuticle melanization and sclerotization are broadly conserved among insects [1, 2]. Briefly, both processes share a common initial pathway that starts in the epidermis with the hydroxylation of l-tyrosine into DOPA by the tyrosine hydroxylase (TH) enzyme, followed by decarboxylation into dopamine by dopa decarboxylase (DDC). Dopamine can then be oxidized into black melanin via phenoloxidases (melanization pathway) or be transformed into N-β-alanyldopamine (NBAD) via an NBAD synthase and enter the sclerotization pathway, which results in the production of quinones. In the cuticle, the quinones catalyze the formation of adducts between cuticular proteins (CPs) and chitin, thereby providing rigidity to the cuticle [3].

The rapid tanning (melanization + sclerotization) of the cuticle that occurs after ecdysis is under the control of the so-called “tanning hormone”, bursicon, which is highly conserved among insects [4–6]. Bursicon is a heterodimeric neurohormone, which acts on the G protein coupled receptor LGR2 [7, 8]. In *Drosophila*, DLGR2 is encoded by the *rickets* gene (*rk*). Flies mutant for *rk* or for bursicon subunits (encoded by the *bursicon* (*burs*) and *partner of bursicon* (*pburs*) genes, also known, respectively, as *bursicon* α and *bursicon* β) fail to tan properly; they also show an altered body shape and do not expand their wings [9, 10]. Bursicon is synthesized by neurons located in the subesophageal ganglion (SEG) and in abdominal ganglia, which also produce the neuropeptide, crustacean cardioactive peptide, CCAP [10]. Once the adult fly emerges from its puparium, bursicon is released into the hemolymph mostly by the neurons of the abdominal ganglia [11, 12]. How bursicon then causes the tanning of the cuticle is unclear. Nevertheless, it has been assumed that it would act directly on the epidermis to cause the secretion of melanin and reactive quinones into the overlying extracellular cuticular matrix [13–15]. Consistent with this hypothesis, *rk* is expressed in epidermal cells, and *rk* mutants show a delay in the phosphorylation of epithelial TH, which is necessary for the activation of the tanning pathway [13].

Here we use a variety of genetic approaches to show that the tanning of the *Drosophila* adult cuticle is not caused by direct actions of bursicon on the epidermis. Instead, we found that RK is required in a small set of peptidergic neurons in the ventral nervous system (VNS) in order for the rapid melanization and sclerotization of the cuticle to occur following adult emergence. Our findings challenge the current view of bursicon action and reveal previously unsuspected elements involved in the pathway that controls the rapid postecdysial maturation of the insect cuticle.

## Results

### Loss of RK function prevents cuticle sclerotization and delays the melanization of the adult fly but eventually results in a darker than normal exoskeleton

**Fig. 1:**
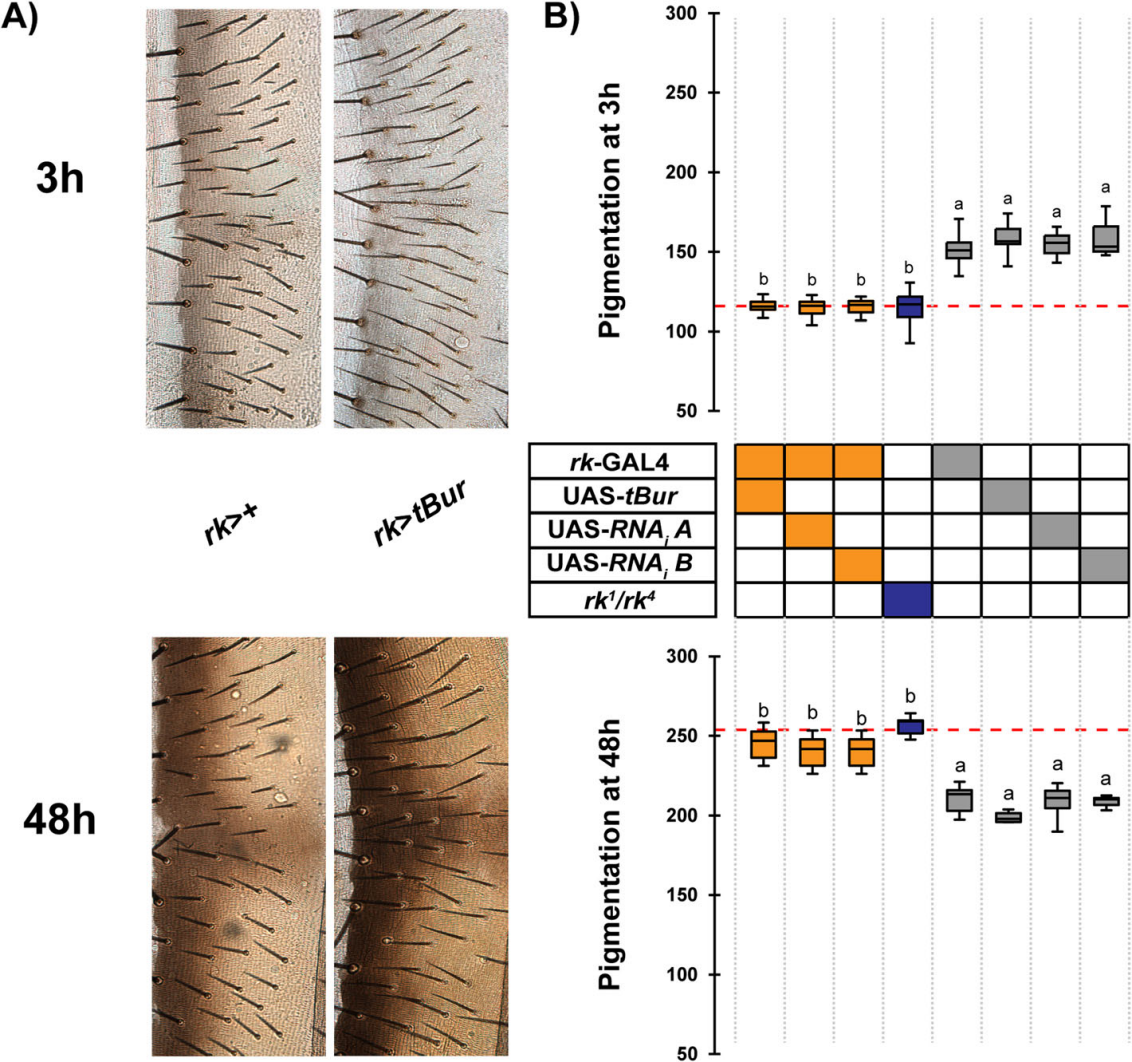
Ubiquitous knockdown of *rk* differentially affects melanization at 3 h and 48 h post emergence. **a** Representative pictures of the fourth abdominal segment of female control (left) and *rk*>*tBur* (right) flies at 3 h (upper panel) and 48 h (lower panel) after emergence. **b** Quantification of abdominal pigmentation measured in 3-h- (upper panel) and 48-h-old (lower panel) female flies expressing *tBur* or two different *rk* RNAi transgenes (RNAi A and RNAi B) under the control of the *rk*-GAL4 driver (*rk*>); in *rk*^*1*^*/rk*^*4*^ mutant flies; and in controls. Genotypes are indicated by the combination of squares within each column, and color-coded as follows: blue squares correspond to mutant animals (in this case *rk*^*1*^*/rk*^4^), orange squares represent transgenic combinations (e.g., *rk*>*tBur*, for the first column), and gray boxes correspond to control genotypes (e.g., GAL4 drivers alone). Boxes mark the first and third quartiles, thick lines mark the medians, and whiskers represent data range. Red dashed line indicates the median pigmentation level of *rk* mutants and will be used as a visual reference in all figures of this type. Results for each age were compared using a one-way ANOVA followed by Tukey HSD post hoc analysis. Different letters indicate statistically significant differences (one-way ANOVA followed by Tukey HSD, *p* < 0.01). *n* = 10 for each group

Flies trans-heterozygous for loss-of-function *rk* mutations (*rk*^*1*^*/rk*^*4*^) do not expand their wings, present an abnormal body shape and fail to melanize properly [9]. A comparable result was obtained when a *rk* RNAi or a membrane tethered bursicon hormone transgene (here called *tBur*), which acts as a dominant negative *rk* allele [16, 17], was expressed in all *rk*-expressing cells using a *rk*-GAL4 driver (Fig. 1a,b; results shown for female flies; similar results were obtained with adult male flies, Additional file 1: Figure S1). In all cases, the resulting adult flies did not expand their wings and their bodies did not rapidly pigment after adult emergence. Indeed, at 3 h posteclosion, median cuticle pigmentation was around 150 for control flies whereas it was around 100 for flies expressing *tBur* under the control of the *rk*-GAL4 driver (− 33%)(see “Methods” section for a description of the method used here to quantify cuticle pigmentation). Interestingly, at 48 h, the flies with impaired RK function showed a significantly darker pigmentation than their respective controls. Indeed, by 2 days posteclosion, the median score for these flies was around 250, whereas it was around 200 for controls (+ 25%). Importantly, these experiments revealed that driving the *tBur* transgene using the *rk-*GAL4 driver (abbreviated here *rk*>*tBur*) phenocopied the pigmentation defects expressed by *rk*^*1*^*/rk*^*4*^, and by *rk*^*1*^*/rk*^*1*^ and *rk*^*4*^*/rk*^*4*^ (not shown), mutant flies. For this reason, we chose to use the *tBur* transgene to interfere with RK function for most experiments reported here.

**Fig. 2:**
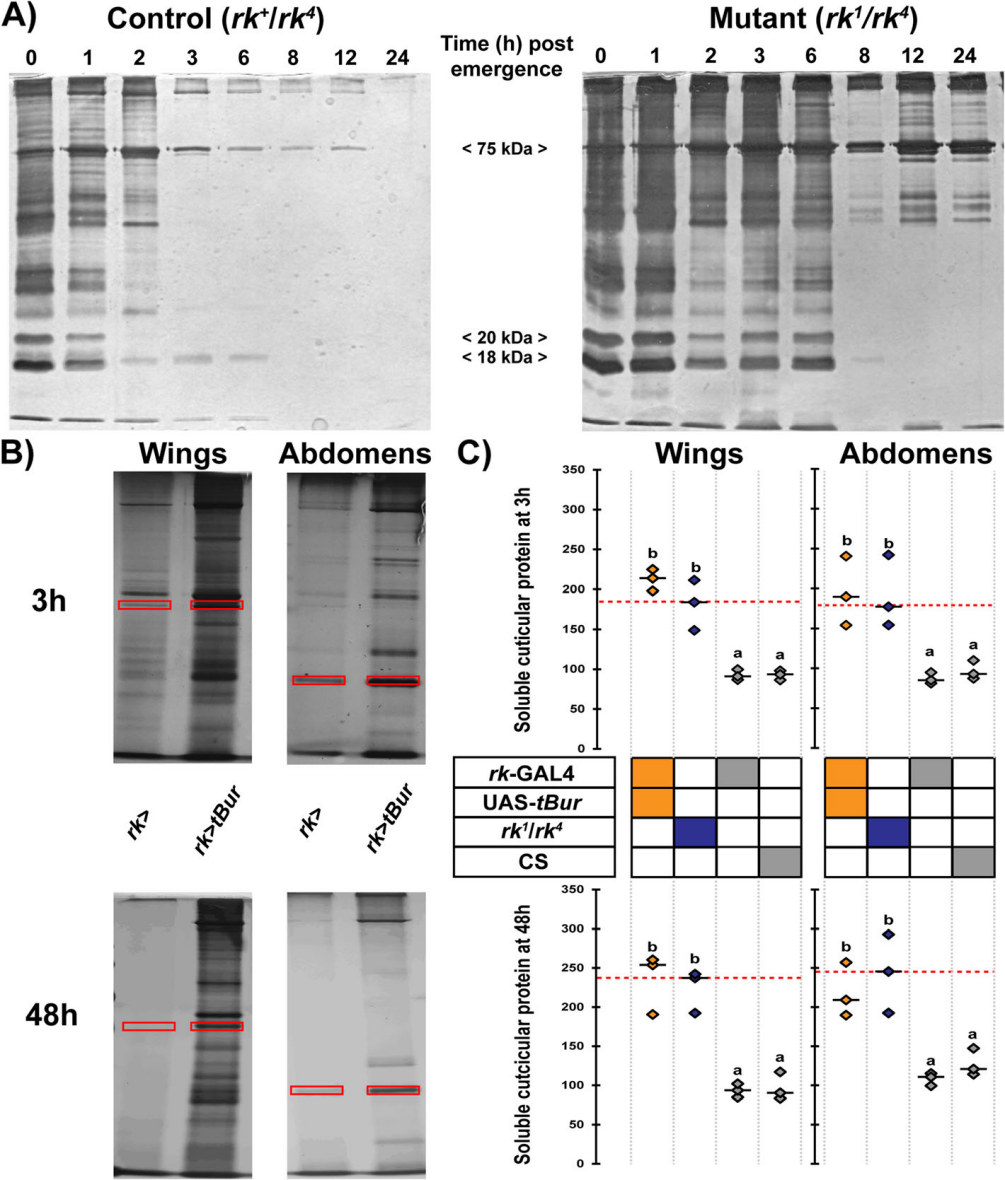
Sclerotization is nearly complete by 3 h in wildtype flies whereas *rk* mutant flies and flies with ubiquitous knockdown of *rk* express defective sclerotization up to 48 h postemergence. **a** Visualization of soluble cuticular proteins extracted from wings at 0 h, 1 h, 2 h, 3 h, 6 h, 8 h, 12 h, and 24 h postemergence in control (heterozygous *rk*^*4*^*/rk*^*+*^ flies, left panel) and in transheterozygous *rk* mutant (*rk*^*4*^*/rk*^*1*^, right panel) flies. **b** Pictures of representative silver-stained gel of soluble cuticular proteins extracted from wings (left) or abdominal epidermis (right) of control (*rk*>+) and *rk* knockdown (*rk*>*tBur)* flies at 3 (upper panel) and 48 (lower panel) hours after emergence. Red rectangles indicate the bands that were quantified in **c**. **c** Intensity of bands marked in **b** for three separate experiments; short black horizontal lines indicate the median. Genotypes are coded as described in Fig. 1b. Different letters indicate statistically significant differences (one-way ANOVA followed by Tukey HSD, *p* < 0.01). For quantification of other bands (indicated in Additional file 1: Figure S2B), see Additional file 1: Figure S5

In addition to defects in the timecourse of melanization, we noticed that, at 48 h posteclosion, both the cuticle shape and its appearance were abnormal in *rk*^*1*^*/rk*^*4*^ flies and when *tBur* was expressed under the control of *rk*-GAL4 driver. Indeed, the abdominal cuticle of these flies showed abnormal folds (Additional file 1: Figure S2A) and also appeared matte compared to that of control flies, which always looked shiny by this time. Since these phenotypes could be caused by defects in sclerotization, we developed a semi-quantitative assay to measure the extent of cuticle hardening, based on the levels of soluble proteins that could be extracted from either the abdominal or the wing cuticle, and visualized in silver-stained protein gels (Fig. 2 and Additional file 1: Figure S2B). This assay showed that the levels of soluble proteins that could be extracted from the cuticle of control flies was maximal at 0 h post eclosion, decreased slightly at 3 h and was almost undetectable at 48 h, indicating that by 2 days posteclosion the protein crosslinking reaction that underlies the sclerotization process had rendered insoluble most cuticular proteins. By contrast, the levels of soluble proteins that could be extracted from *rk*^*1*^*/rk*^*4*^ and *rk*>*tBur* flies showed a twofold increase at 3 h compared to those of their respective control and many proteins remained detectable even 48 h after emergence.

### Bursicon does not act directly on the epidermis to induce cuticle tanning

**Fig. 3:**
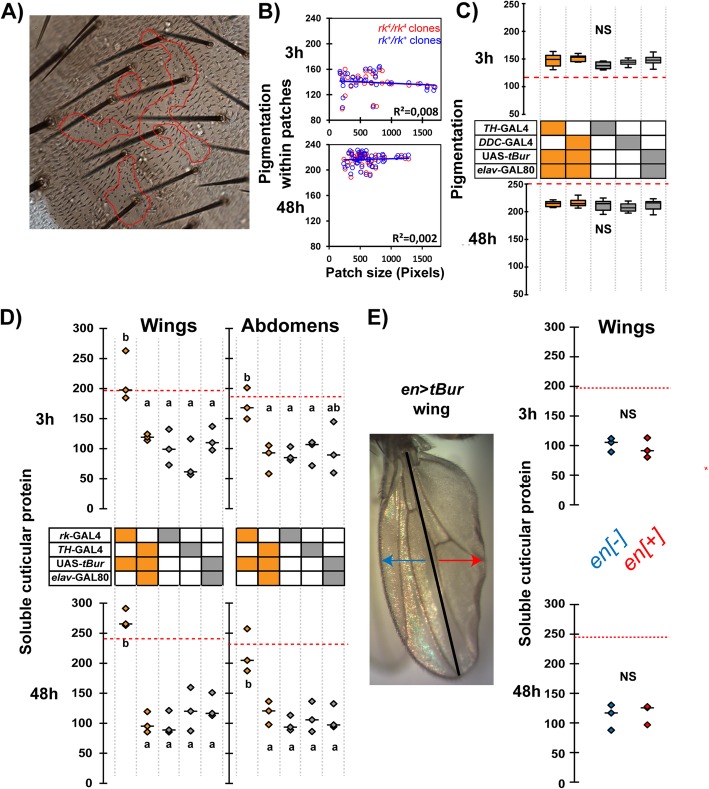
Bursicon does not act directly on the epidermis to regulate tanning. **a** Abdominal cuticle of 3-h-old mosaic fly bearing patch of *rk*^*4*^*/rk*^*4*^ mutant tissue in an otherwise *rk*^*4*^*/rk*^*+*^ animal. Mutant tissue is marked by the cell-autonomous *stc* mutation and is outlined in red. **b** Quantification of pigmentation of cuticle overlying *rk*^*4*^*/rk*^*4*^ epidermal clones (red circles) or *rk*^*+*^*/rk*^*+*^ control clones (blue circles) as a function of clone size. Neither clone size nor genotype effected significantly the pigmentation at 3 h or 48 h postemergence (ANCOVA analysis; *n* ≥ 35 clones from 15 flies per group). **c** Abdominal pigmentation of flies in which *tBur* expression was driven in the epidermis using the *TH*-GAL4 and *DDC*-GAL4 drivers in combination with *elav*-GAL80 (to restrict expression to the epidermis). *n* = 10 for each group. Genotypes are coded as described in Fig. 1b. NS: not significantly different (one-way ANOVA, *p* > 0.56; *n* = 10 per group). **d** Quantification of soluble cuticular proteins extracted from wings and abdominal epidermis of flies expressing *TH*>*tBur* in combination with *elav*-GAL80, in *rk>tBur* flies, and in their respective controls. Genotypes are coded as described in Fig. 1b. Intensity of reference bands (cf. Fig. 2b) is shown for three separate experiments; median is indicated by short horizontal line. Different letters indicate statistically significant differences (one-way ANOVA followed by Tukey HSD, *p* < 0.001). For quantification using other bands (indicated in Additional file 1: Figure S2B), see Additional file 1: Figure S6. **e** Left: image of wing of *engrailed*>*tBur* (*en*>*tBur*) fly. Right: Quantification of soluble cuticular proteins extracted from the posterior and anterior half of the wing. Although the posterior half of the wing appears misfolded, the amounts of soluble protein extracted from each half did not differ significantly (measurements shown for three separate experiments; median is indicated by short horizontal line). NS: non-significant (paired *t*-test, *p* > 0.7)

To determine if bursicon acts directly on the epidermis to cause melanization, we created mosaic flies bearing marked patches of homozygous *rk* mutant (*rk*^*4*^/*rk*^*4*^) epidermal cells in an otherwise normal (*rk*^*4*^/*rk*^*+*^) animal. Surprisingly, the cuticle overlying the patches of homozygous *rk* mutant epidermis did not show any pigmentation defects at 3 h or 48 h post emergence (Fig. 3a,b); this result was observed regardless of patch size, consistent with the cell-autonomous property expected of the *rk* GPCR. To confirm this result using a separate approach, we expressed *tBur* in the epidermis using two different epidermal GAL4 driver lines: *Tyrosine Hydroxylase*-GAL4 (*TH*-GAL4) and *Dopa-Decarobylase*-GAL4 (*DDC*-GAL4), both used in combination with *elav*-GAL80 to prevent knockdown of RK expression in the nervous system. Consistent with the results obtained using mosaic animals, we found that knockdown of RK function in the epidermis produced flies with normal levels of pigmentation at 3 h and 48 h post emergence (Fig. 3c; results shown for female flies; similar results were obtained in males, Additional file 1: Figure S1). Similar results were obtained using *rk* RNAi transgenes driven by *TH*-GAL4 (Additional file 1: Figure S3). Taken together, these results show that RK function is not required in the epidermis to regulate postecdysial melanization.

We noticed that the cuticle of flies in which RK function had been knocked down in the epidermis showed abnormal folds and a matte appearance, suggesting that in the epidermis RK may be involved in the process of sclerotization. To address this hypothesis, we estimated the quantity of soluble protein present in abdominal or wing cuticle of flies in which RK function was knocked down in the epidermis. Surprisingly, neither wings nor abdomens showed any increase in the levels of soluble proteins in these flies compared to those of their respective control (Fig. 3d). In parallel experiments, we used the *engrailed-GAL4* (*en*-GAL4) driver to express *tBur* in the posterior half of the adult wing. Although the posterior part of the wings appeared misfolded and more matte than its anterior counterpart (Fig. 3e, left panel), we found no differences in the amounts of soluble protein that could be extracted from the anterior vs. the posterior half of the wing at either 3 h or 48 h post emergence (Fig. 3e, right panel). Taken together, these results show that RK function is not required in the epidermis to regulate postecdysial melanization or sclerotization. Nevertheless, the expression of *rk* in the epidermis and the morphological defects observed when *RK* function is disabled in the epidermis (e.g., Fig. 3e, left panel; Additional file 1: Figure S2A) suggest that *rk* may play an additional, if currently unknown, role in this tissue.

### *rickets* function is required in the CNS to regulate postecdysial cuticle maturation

**Fig. 4:**
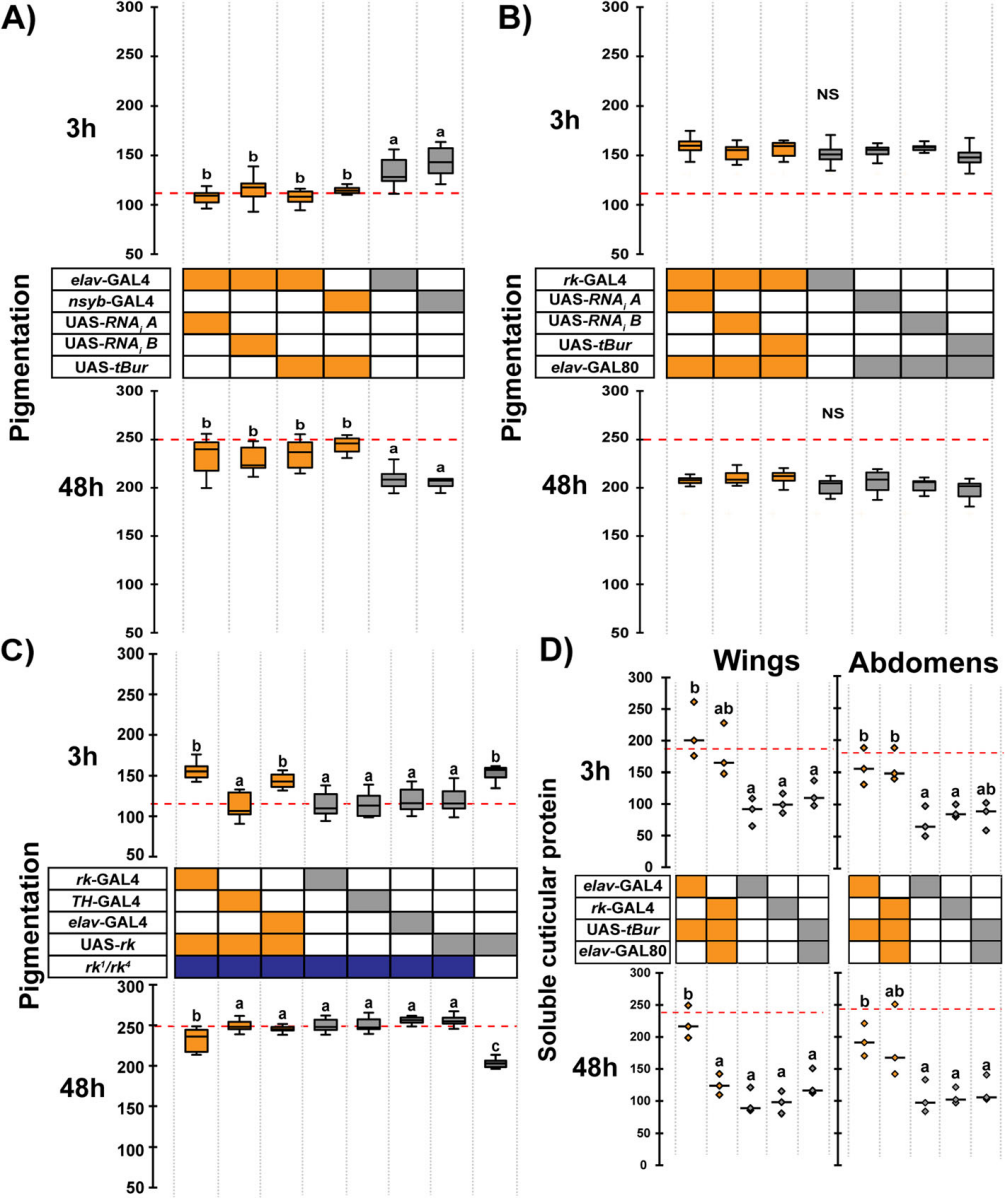
Bursicon acts on *rk*-expressing neurons to cause melanization and sclerotization. **a** Abdominal pigmentation in 3-h- (upper panel) and 48-h-old (lower panel) female flies expressing *tBur* or *rk* RNAi transgenes under the control of pan-neuronal drivers (*elav*-GAL4 and *nsyb*-GAL4). **b** Abdominal pigmentation in 3-h- (upper panel) and 48-h-old (lower panel) female flies expressing *tBur* or *rk* RNAi transgenes under the control of *rk*-GAL4 and restricted to non-neuronal *rk* cells using *elav*-Gal80. **c** Rescue of abdominal pigmentation in 3-h- (upper panel) and 48-h-old (lower panel) female *rk*^*1*^*/rk*^4^ flies expressing a *rk* cDNA [19] under the control of the *rk*-GAL4, *TH*-GAL4, and *elav*-GAL4, drivers. In **a–c**, boxes indicate the first and third quartiles, thick central lines mark the medians, and whiskers represent data range. Red dashed lines indicate the median pigmentation level when *tBur* is expressed ubiquitously (*rk>tBur*). Results for each age were compared using a one-way ANOVA followed by Tukey HSD post hoc analysis. Different letters indicate statistically significant differences (*p* values < 0.01); NS: not significantly different. *n* = 10 for each group. **d** Quantification of soluble cuticular proteins extracted from wings and abdominal epidermis of *elav*>*tBur*, *rk*>*tBur* female flies with or without *elav*-GAL80, in three separate experiments; median is indicated by short horizontal line. For quantification of other proteins (indicated in Additional file 1: Figure S2B) see Additional file 1: Figure S7. Different letters indicate statistically significant differences (one-way ANOVA followed by Tukey HSD, *p* < 0.02). Genotypes are coded as described in Fig. 1b

**Fig. 5:**
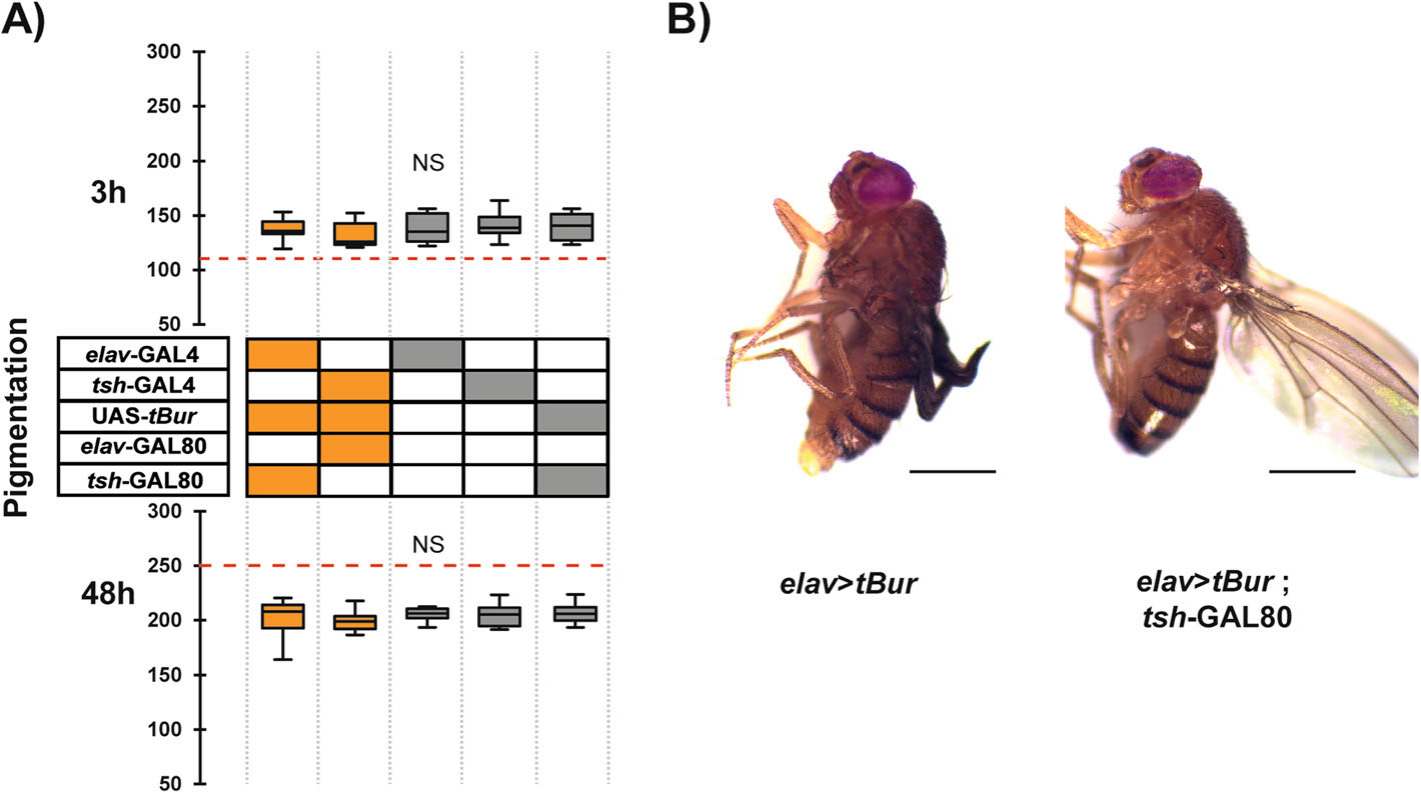
*rk*-expressing neurons required for the control of pigmentation are located in the VNS. **a** Abdominal pigmentation in 3-h- (upper panel) and 48-h-old (lower panel) female flies expressing *tBur* under the control of pan-neuronal driver, *elav*-GAL4, whose expression was restricted to the brain using *tsh*-GAL80. Genotypes are coded as described in Fig. 1b. Boxes indicate the first and third quartiles, thick central lines mark the medians, and whiskers represent data range. Red dashed line indicates the median pigmentation level when *tBur* is expressed ubiquitously (*rk*>*tBur*). Results were compared using a one-way ANOVA for each age group and found to not be statistically different (NS). *n* = 10 for each group. **b** Representatives pictures of 48-h-old females. Left: *elav*>*tBur* fly displaying wing expansion failure and a darker abdomen. Right: *elav*>*tBur*; *tsh*-GAL80 fly displaying normal pigmentation and wings. Scale bar = 1 mm

The *rk* gene is widely expressed in the CNS (see Additional file 1: Figures S9 and S10, below), and *rk*-expressing neurons have recently been shown to play a critical role during pupal ecdysis [18]. To investigate a potential role for *rk* in the CNS for cuticle darkening, we first examined the pigmentation of flies in which *rk* was knocked down in the CNS by driving *tBur* using the GAL4 drivers, *elav*-GAL4 and *nsyb*-GAL4 (which are known to drive gene expression in the CNS and not in the epidermis). As shown in Fig. 4a, these flies showed pigmentation defects at both 3 h and 48 h postemergence, which were similar to those expressed by *rk*^*1*^/*rk*^*4*^ mutant flies. Comparable results were obtained using these GAL4 lines to drive *rk* RNAi transgenes (Fig. 4a). In addition, the wings of these flies failed to expand (Fig. 5b), which is expected because wing expansion requires the nervous system to cause the abdomen to contract and pump hemolymph into the wings [9]. Importantly, the pigmentation (Fig. 4b) and wing expansion defects (not shown) expressed by *rk*>*tBur* flies were rescued when combined with *elav*-GAL80, confirming that cuticle melanization and wing expansion require RK function in the CNS. Conversely, driving a *rk* cDNA using the neuron-specific driver, *elav*-GAL4, in *rk*^*1*^*/rk*^*4*^ mutant flies rescued pigmentation at 3 h (Fig. 4c, top), indicating that restoring *rk* function in the CNS is sufficient to cause normal cuticle melanization at this time. This contrasts to the results obtained using the (primarily) epidermal driver, *TH*-GAL4, for which no such rescue was obtained. Intriguingly, no rescue was obtained at 48 h post emergence using the *elav*-GAL4 driver (Fig. 4c, bottom). Nevertheless, only partial rescue was obtained at this time using the *rk*-GAL4 driver (which does rescue wing expansion ([19], and data not shown), suggesting that the UAS-*rk* construct may not provide wildtype levels of RK function regardless of the GAL4 driver used.

Interestingly, *elav*>*tBur* flies exhibited postemergence sclerotization defects in both wings and abdomens, thereby also implicating RK function in neurons in this process (Fig. 4d). Surprisingly, and in contrast to what occurred for melanization, including the *elav*-GAL80 transgene in *rk*>*tBur* flies was not sufficient to rescue the sclerotization defect except in 48 h wings. Thus, these results suggest that RK function in the CNS is necessary but not sufficient to control cuticle sclerotization.

### *rickets* function is required in the ventral nervous system to regulate postecdysial pigmentation

We then used different GAL4 drivers to pinpoint the *rk* neurons that could be the direct targets of bursicon involved in the control of cuticle melanization. To this end, we first knocked down RK function only in brain neurons by driving *tBur* expression using the pan-neuronal *elav*-GAL4 driver in combination with *tsh*-GAL80, which drives GAL80 (thereby inhibiting GAL4) expression in the trunk [20]. As shown in Fig. 5a, these flies did not exhibit pigmentation defects at either 3 h or 48 h post emergence, and wing expansion was also normal in these flies (Fig. 5b). Conversely, when we then drove *tBur* only in the VNS using a *tsh*-GAL4 driver most the flies died at the start of metamorphosis yet the rare escapers exhibited a *rk* mutant phenotype (not shown). Taken together, these results show that RK function is primarily required in the VNS for the rapid postemergence melanization of the cuticle. Interestingly, we were able to rescue the pupal lethality observed in *rk*>*tBur* animals by including the *elav*-GAL80 transgene, consistent with the known role for *rk* signaling during pupal ecdysis [18, 21].

### *rickets* function is required for cuticle melanization in peptidergic neurons that are not the neurons that produce bursicon

**Fig. 6:**
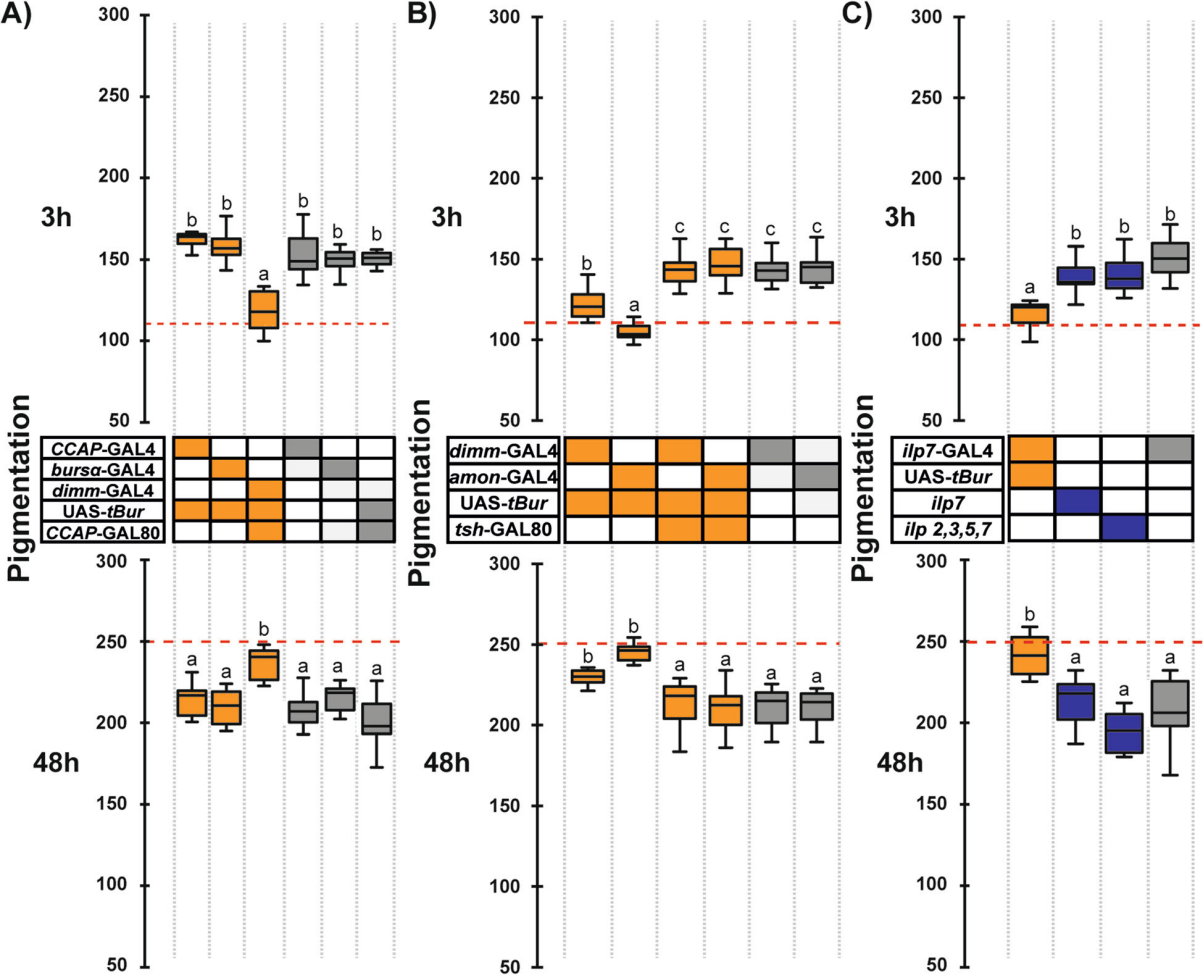
Peptidergic *rk*-expressing neurons participate in the control of melanization. **a** Abdominal pigmentation in 3-h- (upper panel) and 48-h-old (lower panel) female flies expressing *tBur* in: bursicon neurons using *CCAP*-GAL4 (which drives expression in all bursicon neurons from the VNS [10]) and *burs*-GAL4 (which drives expression in all bursicon neurons [11]); and in peptidergic neurons using the *dimm*-GAL4 driver and restricted to non-CCAP neurons using CCAP-GAL80. **b** Abdominal pigmentation in 3-h- (upper panel) and 48-h-old (lower panel) female flies expressing *tBur* under the control of peptidergic neuron drivers (*dimm*-GAL4 and *amon*-GAL4) and restricted to VNS using *tsh*-GAL80. **c** Abdominal pigmentation in 3-h- (upper panel) and 48-h-old (lower panel) female flies expressing *tBur* in ILP7 neurons using *ilp7*-GAL4; in *ilp7* null mutants; and in flies (multiply) mutant for *ilp2*, *ilp3*, *ilp5*, and *ilp7*. Genotypes are coded as described in Fig. 1b; boxes indicate the first and third quartiles, thick central lines mark the medians, and whiskers represent data range. Red dashed lines indicate the median pigmentation level when *tBur* is expressed ubiquitously (*rk*>*tBur*). *n* = 10 in each group. Results for each age were compared using a one-way ANOVA followed by a Tukey HSD post hoc analysis. Different letters indicate statistically significant differences (*p* values ≤ 0.02 for **a**; *p* ≤ 0.01 for **b** and *p* ≤ 0.0001 for **c**). Results for males are presented in Additional file 1: Figure S8

Our results suggest that *rk* is required in the VNS to cause cuticle melanization and wing expansion. A previous study showed that bursicon release is delayed in *rk*^*4*^/*rk*^*4*^ mutant flies [11], suggesting that cuticle melanization might require RK function in the bursicon-secreting neurons themselves. To test this hypothesis, we expressed the *tBur* transgene in bursicon neurons using two different GAL4 drivers: CCAP-GAL4, which drives expression in all CCAP neurons (including all bursicon neurons in the adult) and *burs*-GAL4, which drives expression in the BURS-expressing neurons [10, 12]. As shown in Fig. 6a, these flies were entirely normal, indicating that postemergence melanization does not require an autocrine RK function in the bursicon neurons.

Since ecdysis involves a number of neuropeptides acting on downstream peptidergic neurons [22, 23], we then considered the possibility that cuticle melanization involved bursicon action on other peptidergic neurons. To address this possibility, we used the *tBur* transgene to knock down RK function in large ensembles of peptidergic neurons using the drivers, *dimm-*GAL4 (*dimmed*-GAL4) and *amon*-GAL4 (*amontillado*-GAL4) which reflect the expression of the transcription factor DIMMED, which is required for peptidergic neuron maturation [24], and of the AMONTILLADO proprotein processing enzyme, PC2 [25], respectively. (Both of these drivers are expressed in peptidergic neurons and we confirmed that they are not expressed in the adult epidermis; Additional file 1: Figure S4). As shown in Fig. 6b, expression of *tBur* using *dimm*-GAL4 caused the melanization defects characteristic of *rk* mutant animals, with lighter and darker pigmentation than normal in 3-h- and 48-h-old flies, respectively. Interestingly, these flies were normal in terms of wing expansion. Expression of *tBur* using *amon*-GAL4 caused pigmentation defects similar to those of *rk* mutants at 3 h and 48 h post emergence but, interestingly, also caused wing expansion failures (not shown), and resulted in flies with matte cuticle similar to that observed in *elav*>*tBur* and *rk*>*tBur* flies. Importantly, the defects observed when RK function was knocked down using these drivers were rescued by including the *elav*-GAL80 (Additional file 1: Figure S9A) and *tsh*-GAL80 (Fig. 6b) transgenes, confirming the localization of the *rk* requirement to the CNS. However, they were not rescued by including the CCAP-GAL80 transgene (Fig. 6a), consistent with the results obtained using the CCAP-GAL4 driver to knock down RK function, and the lack of co-expression of CCAP and *rk*-GAL4 in the VNS (Additional file 1: Figure S9B). Taken together, these results suggest that bursicon acts in paracrine rather than an autocrine manner to control cuticle pigmentation.

Downregulation of the insulin receptor has been shown to severely decrease cuticle pigmentation in *Drosophila* [26] suggesting that some insulin-like peptides (ilp) could participate in the regulation of this process. The only ilp known to be expressed in the VNS is ilp7 [27, 28], and we observed that at least two ilp7-immunoreactive neurons coexpress *rk* (Additional file 1: Figure S10). Thus, in a final attempt to identify peptidergic neurons in the VNS that could play a direct role in pigmentation, we expressed *tBur* using an *ilp7*-GAL4 driver and observed significant pigmentation defects at both 3 h and 48 h of age (Fig. 6c). Nevertheless, we did not observe any pigmentation defects in a null mutant allele for *ilp7* or in flies simultaneously mutant for *ilp genes ilp2*, *ilp3*, *ilp5*, and *ilp7* (Fig. 6c). These results suggest that some of the 20 ILP7-secreting neurons from the VNS may be direct targets of bursicon and participate in the regulation of cuticle pigmentation but that this role is not mediated by the ILP7 hormone itself, similarly to what has been reported for fertility regulation [29].

## Discussion

Cuticle tanning encompasses two distinct molecular processes, melanization and sclerotization, which result, respectively, in the darkening and the hardening of the insect cuticle. Though both processes share the same initial molecular steps in the epidermis, they then diverge into two separate pathways [2, 30]. The rapid tanning that occurs after emergence has been known to be under control of a humoral factor for decades, and we also now know the molecular identity of bursicon, the key neurohormone involved in this process, and that of its receptor, DLGR2, encoded in *Drosophila* by the *rickets* (*rk*) gene [4, 6–9]. Mutations in the genes encoding bursicon subunits or in *rk* induce similar defects including a failure in wing expansion and a delay in tanning. Here, we have provided a more comprehensive description of the tanning defects of *rk* mutants and show that although pigmentation levels are lower than normal during the first few hours after emergence, they continue to increase during the following 2 days, such that by 48 h post emergence the flies display an overpigmentation of their abdominal cuticle. This contrasts to normal flies, in which the melanization process is essentially complete by 6 h after emergence. A potential explanation for the overpigmentation of *rk* mutant flies could be that the lack of sclerotization of the cuticle results in an overabsorption of pigments. Consistent with this hypothesis, we recently showed that the cuticle of *rk* mutant flies contain higher levels of cuticular hydrocarbons [31]. Alternatively, the lack of sclerotization could result from a higher tendency of the cuticle to form microfolds (see Additional file 1: Figure S2A), which might increase its opacity.

In addition, we developed here a semi-quantitative assay to evaluate the level of sclerotization of cuticular proteins and show that the cuticle of *rk* mutants contain soluble proteins during at least the first 2 days of adult life, thereby showing that bursicon and RK are involved in the crosslinking of cuticular proteins that underlies the process of sclerotization.

Although the reactions involved in cuticle tanning are well known, how bursicon causes this process to occur rapidly following emergence is still unclear. A key element for understanding this process is identifying where bursicon acts, and it has always been assumed that this hormone acts directly on the epidermis to induce the melanization and sclerotization of the overlying cuticle, which is consistent with the fact that *rk* is expressed in the epidermis in various insect species [19, 32, 33]. Yet, using a variety of genetic techniques, we were surprised to discover that bursicon does not act directly on the epidermis to cause cuticle melanization and sclerotization. Instead, we found that both of these actions are indirect and that melanization is mediated by peptidergic neurons in the ventral nervous system. Although we were ultimately unable to identify the molecular intermediary, our findings suggest that neurons expressing *Ilp7* play a key role in this process, although the ILP7 hormone itself is not involved.

Our results challenge the classical view of bursicon action on the epidermis to regulate cuticle tanning. They also reveal unsuspected complexities in RK action. For instance, although normal melanization and wing inflation require RK function in the CNS, these functions are separable. Indeed, as shown in Table 1, whereas *rk* knockdown using an *amon*-GAL4 driver affected both wing inflation and cuticle melanization, knockdown using the *dimm*-GAL4 driver affected melanization but produced adults with normal wings. Similarly, whereas RK function in the CNS is necessary and sufficient to cause melanization, tissues outside of the CNS (and of the epidermis) are also involved in sclerotization. These tissues are currently unknown, but they do not include muscles, tendon cells, or hemocytes (see Table 1). Our results also raise the obvious question: what is the function of RK in the epidermal cells? Although we show that RK is not required in the epidermis for melanization or sclerotization, *rk* knockdown in the epidermis caused the cuticle to show microfolds (Additional file 1: Figure S2A), a matte finish (Fig. 3e and Table 1), and also caused the wings to be slightly misshapen (Fig. 3e), suggesting that in the epidermis bursicon might affect chitin organization and/or the proportion of given cuticular proteins [34].

**Table 1:** Postemergence maturation defects associated with disabling RK function in different cell types

Tissue targeted^1^	Driver^2^	Pigmentation defects^3^	Sclerotization defects^4^	Wing expansion defects^5^	Cuticle appearance^6^
RK cells	*rk*-GAL4	+++ ^7^	+++	+++	+++
Epidermis	TH-GAL4	–	–	–	+++
Tendon cells	*sr*-GAL4	–	–	–	–
Hemocytes	*hem*-GAL4	–	–	–	–
Muscles	*C59*-GAL4	–	–	–	–
Oenocytes	*desat(RE*)-GAL4	–	–	–	–
Neurons	*elav*-GAL4	+++	+++	+++	+++
	*nsyb*-GAL4	+++	ND	+++	+++
Peptidergic neurons	*amon*-GAL4	+++	ND	+++	++
	*dimm*-gal4	++	ND	–	–
ILP7 neurons	*ilp7*-GAl4	++	ND	–	–

^1^Tissues in which RK function was disabled by driving *tBur*

^2^GAL4 driver used to drive UAS-*tBur* in desired tissues

^3–6^ Defects observed in pigmentation ^3^; sclerotization ^4^ (assessed measuring soluble protein present in cuticle); in wing expansion ^5^ and in cuticle appearance ^6^ (incomplete maturation causes cuticle to have a surface that is matte in appearance)

^7^ Defects were classified qualitatively as severe (+++; similar to those of *rk* mutant animals) to normal (−). *ND* not determined

## In conclusion

Our findings reveal that cuticle tanning is a complex process and challenges the classical view of a direct action of bursicon on the epidermis. We show that RK is required in the VNS for the rapid melanization and sclerotization that occurs after emergence and that these two processes likely require the action of bursicon on different targets and may involve targets outside of the CNS (and the epidermis). Future progress in understanding how the cuticle is pigmented and hardened will now require identifying the intermediaries that transmit to the epidermis the signal provided by the release of bursicon.

## Methods

### *Drosophila* stocks

Flies were raised on standard cornmeal/molasses/yeast food and maintained at 22 °C under a 12 h:12 h light:dark regime. All crosses were performed at 25 °C under 12 h:12 h light:dark regime. Unless noted, fly stocks were obtained from the *Drosophila* Bloomington stock center (BL; Bloomington, USA) and the Kyoto Stock Center (NIG; Kyoto, Japan): *rk*^*1*^ (BL3589) and *rk*^*4*^ (BL3590) alleles; flies mutant for *ilp7* (BL30887), flies mutant for *ilp2*, *ilp3*, *ilp5*, and *ilp7* (BL30893); UAS-RNAi *rk* (NIG8930-R1 [called here RNAi A] and NIG8930-R2 [called here RNAi B]), *elav*-GAL4 (BL8765), *nsyb*-GAL4 (BL51941), *amon*-GAL4 (BL25410), *dimm*-GAL4 (BL25373), *hemese*-GAL4 (BL8699), *sr*-GAL4 (BL2663), C57-GAL4 (BL32556), *pnr*-GAL4 (BL3039), 20XUAS-FLP (BL55805), *hs*-FLP (BL55805). *elav*-GAL80 was obtained from O. Schafer; *nsyb*-GAL80 was obtained from J. Simpson; *rk*-GAL4, UAS-*rk*, and CCAP-GAL80 were provided by B. White [19]; UAS-*tBur* was provided by A. Kopin; and *tsh*-GAL80 was obtained from C. Wegener. Desat(RE)-GAL4 was provided by J.-F. Ferveur; *stc*, FRT39 flies were obtained from G. Struhl; and *ilp7*-GAL4 was obtained from Y. N. Jan.

### Induction of marked *rk* clones

To produce mosaic animals, we relied on a stock that carries the *stc* mutation (a mutant allele of the *crinkled* gene that causes cell-autonomous defects in cuticular microchaetes; [35, 36]), and an FRT site at cytological position 39. The *rk*^*4*^ allele was recombined onto this chromosome using standard genetic techniques. Patches in random locations were produced using a heat shock-driven FLP transgene, whereas larger patches confined to a strip along the dorsal abdomen were induced using a *pnr*-GAL4 driver in combination with UAS-*flp*. Patches of marked *rk*^*4*^ mutant tissues were created in *rk*^*4*^, *stc*, FRT39/*rk*^*+*^, FRT39 flies, whereas patches of wildtype tissues (controls) were induced in *rk*^*+*^, *stc*, FRT39/*rk*^*+*^, FRT39 flies.

### Measurements of abdominal pigmentation

Three- and 48-h-old flies were frozen at − 20 °C and kept at this temperature until dissection. Flies were dissected under PBS and their abdomens fixed in 4% buffered paraformaldehyde for 1 h, then rinsed and mounted in glycerol, and kept at 4 °C. Images were acquired at × 20 magnification using a Leica DFC480 camera under white light. We quantified melanization as a mean gray value (MGV) using the NIH ImageJ software [37]. Measurements were taken at the fourth segment (A4) for all experiments. Two measurements per fly were taken in this segment, one in band of pigmentation at the posterior edge of the segment, and one in the upper (lightly pigmented) part of the segment (see Additional file 1: Figure S2A for more details). All preparations for a given experiment were quantified during a single session, using the same settings. Microscope lighting conditions were set to produce readings that usually ranged between 15 (most melanized cuticle) to 220 (least melanized). A melanization score for each fly was produced by averaging the 2 readings for each segment. In addition, in order to produce a more intuitive score for which darker cuticles had larger values, the final score was obtained by subtracting this average from 300. Thus, the score was obtained using the following formula:

$$ \mathrm{Pigmentation}=-\left(\frac{\mathrm{MGV}\ \mathrm{black}\ \mathrm{strip}+\mathrm{MGV}\ \mathrm{brown}\ \mathrm{strip}}{2}\right)+300 $$

The value of 300 was chosen arbitrarily because it was the smallest value that produced positive values for all readings.

For mosaics, pigmentation was measured within patches as described above but one measurement was taken from one to three clones per fly, in at least 15 different flies.

### Measurements of wing and abdomen sclerotization

Three- and 48-h-old females were frozen at − 20 °C and kept at this temperature until dissection. They were then dissected in PBS and the wings and the abdominal cuticle were separated and kept at − 80 °C for 24 h before protein extraction. Five abdomens or 5 pairs of wings were vortexed for 15 min at room temperature using tungsten balls in 100 μL of extraction buffer containing Tris (1%, pH = 7.2), 1% SDS, and cOmplete protease inhibitor, EDTA-free, EASYpack (a general cocktail of protease inhibitors; Roche, USA). After a centrifugation, 40 μL of supernatant were mixed with 10 μL of protein loading buffer and 15 μL were loaded on a 10% polyacrylamide gel and run at 110 V. Fifteen microliters of a reference extract was run as a common internal control for all experiments in order to ensure reproducibility of the staining protocol across gels. This reference extract was made as described above but using 100 abdomens or 200 wings of *w*^*1118*^ control flies and aliquoted and kept at − 20 °C. Gels were fixed overnight in 10% acetic acid and 30% ethanol solution and processed through a fast silver staining protocol [38]. Prominent bands were analyzed using ImageJ software. We assessed band intensity measuring its MGV and subtracting background MGV; intensity was then expressed as a percentage of the intensity measured for the reference control lane (see Additional file 1: Figure S2 for more details). Results obtained using the most conserved band are shown in the main figures; results for other bands are shown in Additional file 1: Figures S5, S6, and S7.

### Immunostaining and imaging

Flies were dissected upon emergence in PBS and their VNS or epidermis was fixed for 1 h in 4% buffered paraformaldehyde. To directly observe the fluorescence from the GFP or RFP reporter, CNSs were rinsed in PBS and mounted on poly-lysine coated slides. Co-labeling for *rk* and CCAP or ILP7 expression was done using *rk*>mCherry flies processed for CCAP or ILP immunoreactivity, using a rabbit anti-CCAP antibody (used at 1:5000; a kind gift from H.-J. Agricola, Jena University, Jena, Germany) or a rabbit anti-ILP7 antibody (used at 1:1000; a kind gift from Ernst Hafen, Institute of Molecular Systems Biology, ETH Zürich, Switzerland), respectively. CCAP- and ILP7-immunoreactivity was visualized using an Alexa 488 conjugated goat anti-rabbit secondary antibody (used at 1:500; Invitrogen, CA, USA). Preparations were examined under a spinning disc microscope (Olympus DSU).

### Assessment of *rk* RNAi knockdown efficiency

Efficiency of *rk* RNAi knockdown when using *TH*-GAL4 and *rk*-GAL4 drivers was assessed by real-time qPCR and is shown in Additional file 1: Figure S11. Total RNA was isolated from epidermis obtained from recently emerged adult flies using Trizol (Ambion, Life Technology) following the manufacturer’s protocol and treated with DNAse I (Fermentas; Thermo Fisher Scientific). Total RNA concentration and purity was estimated using a Qubit spectrophotometer (Thermo Fisher Scientific). cDNAs were synthesized using Super Script II reverse transcriptase (Thermo Fisher Scientific). All cDNA samples were standardized to 12.5 ng of total RNA equivalent per μL. *rk* RNA expression was estimated using qPCR and Maxima SYBR Green/ROX qPCR Master Mix (Thermo Fisher Scientific) in a Strategene Mx3000P Real-Time qPCR System (Agilent Technologies). The quantification of gene expression was made using the *rp49* as a housekeeping gene. The primers used for qPCR were:

For *rk*

rk-qF1: CTGCGGCAGAGAAGTGAGTG and

rk-qR1: CGCTGTCGTCGCTTTTGTTG

For *rp49*:

rp49-qF1: ATCTGATGCCCAACATCGGTTA and

rp49-qR1: CACGTTGTGCACCAGGAACTT.

### Statistics

Pigmentation measurements are shown using boxplots representing the median, the 1st and 3rd quartiles, and maxima/minima. Statistically significant differences were determined using one-way ANOVA followed by a Tukey HSD post hoc analysis when samples were normally distributed or by a Kruskal Wallis test followed by Conover-Iman post hoc analysis when strong deviation from normality was detected (Shapiro test and QQ plot). In experiments involving mosaic animals (cf., Fig. 3a,b), an ANCOVA analysis was performed to determine the effect of clone size and genotype on clone pigmentation. All statistical analyses were performed using XLSTAT 2016©. Soluble protein quantification is shown as a simple dot plot with median indicated.

## Supplementary information

Additional file 1: **Figure S1.** Pigmentation of abdomen in males for which RK function has been downregulated ubiquitously, or specifically in the epidermis or in the CNS. **Figure S2.** Details of methodology used to quantify abdominal cuticle pigmentation and sclerotization. **Figure S3.** *rk* knockdown in the epidermis does not induce pigmentation defects. **Figure S4.** Epidermal expression of GAL4 drivers used in this study. **Figure S5.** Sclerotization, quantified using other protein bands, in *rk* mutant females and in females expressing ubiquitous knockdown of RK function. **Figure S6.** Sclerotization, quantified using other protein bands, showing that RK function is not required in the epidermis to regulate sclerotization. **Figure S7.** Sclerotization, quantified using other protein bands, show that RK function is necessary but not sufficient in the CNS to regulate sclerotization. **Figure S8.** Abdominal pigmentation in males in which RK function was downregulated in peptidergic, CCAP, and ILP7 neurons. **Figure S9.** RK function is necessary in peptidergic neurons that are not CCAP immunopositive to regulate melanization. **Figure S10.** Some ILP7-immunopositive neurons express *rk*. **Figure S11.** *rk* knockdown efficiency. (PDF 10369 kb)

## Data Availability

All data generated or analyzed during this study are included in this published article and its supplementary information files.
