# Supplementary material for: The tanning hormone, bursicon, does not act directly on the epidermis to tan the Drosophila exoskeleton
Source: BMC Biol. 2020 Feb 19;18:17. doi: 10.1186/s12915-020-0742-5 (PMC7029472; doi:10.1186/s12915-020-0742-5)
Supplement: Supplementary file 1 — Figure S1. Pigmentation of abdomen in males for which RK function has been downregulated ubiquitously, or specifically in the epidermis or in the CNS. Figure S2. Details of methodology used to quantify abdominal cuticle pigmentation and sclerotization. Figure S3. rk knockdown in the epidermis does not induce pigmentation defects. Figure S4. Epidermal expression of GAL4 drivers used in this study. Figure S5. Sclerotization, quantified using other protein bands, in rk mutant females and in females expressing ubiquitous knockdown of RK function. Figure S6. Sclerotization, quantified using other protein bands, showing that RK function is not required in the epidermis to regulate sclerotization. Figure S7. Sclerotization, quantified using other protein bands, show that RK function is necessary but not sufficient in the CNS to regulate sclerotization. Figure S8. Abdominal pigmentation in males in which RK function was downregulated in peptidergic, CCAP, and ILP7 neurons. Figure S9. RK function is necessary in peptidergic neurons that are not CCAP immunopositive to regulate melanization. Figure S10. Some ILP7-immunopositive neurons express rk. Figure S11. rk knockdown efficiency. (PDF 10369 kb) [file 12915_2020_742_MOESM1_ESM.pdf]

TITLE

The tanning hormone, bursicon, does not act directly on the epidermis to tan the *Drosophila* exoskeleton.

Justin Flaven-Pouchon[1], Javier V. Alvarez[1], Candy Rojas[1], and John Ewer[1, 2]

[1] Instituto de Neurociencia, Universidad de Valparaíso, Valparaiso, Chile

[2] Corresponding author. Email: john.ewer@uv.cl

ADDITIONAL FILES

Supplementary Figures S1-S11

Figure S1. Pigmentation of abdomen in males for which RK function has been downregulated ubiquitously, or specifically in the epidermis or in the CNS.

Figure S2. Details of methodology used to quantify abdominal cuticle pigmentation and sclerotization.

Figure S3. *rk* knockdown in the epidermis does not induce pigmentation defects.

Figure S4. Epidermal expression of GAL4 drivers used in this study.

Figure S5. Sclerotization, quantified using other protein bands, in *rk* mutant females and in females expressing ubiquitous knockdown of RK function.

Figure S6. Sclerotization, quantified using other protein bands, showing that RK function is not required in the epidermis to regulate sclerotization.

Figure S7. Sclerotization, quantified using other protein bands, show that RK function is necessary but not sufficient in the CNS to regulate sclerotization.

Figure S8. Abdominal pigmentation in males in which RK function was downregulated in peptidergic, CCAP, and ILP7 neurons.

Figure S9. RK function is necessary in peptidergic neurons that are not CCAP immunopositive to regulate melanization.

Figure S10. Some ILP7-immunopositive neurons express *rk*.

Figure S11. *rk* knockdown efficiency.

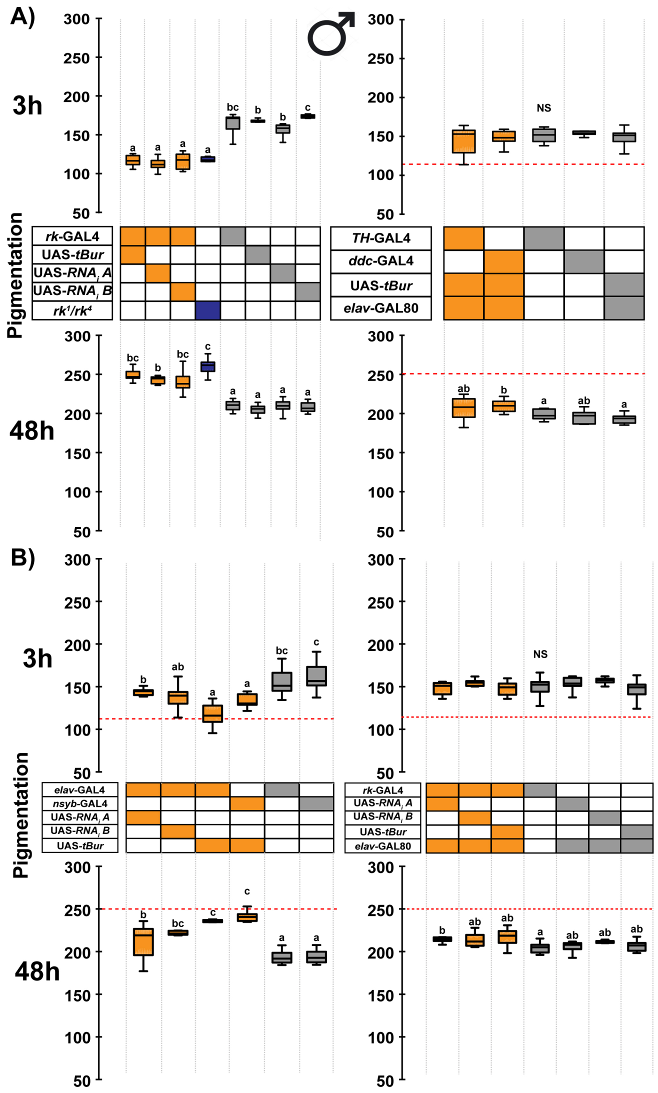

Figure S1. Pigmentation of abdomen in males for which RK function has been downregulated ubiquitously, or specifically in the epidermis or in the CNS. (A) Left panel: Quantification of abdominal pigmentation measured in 3h- (upper panel) and 48h-old (lower panel) male flies expressing *tBur* or 2 different *rk* RNAi transgenes (RNAi A or RNAi B) driven by the *rk*-GAL4 driver (*rk*>); in rk^1/rk^4 mutants; and in controls. Right panel: Abdominal pigmentation of male flies in which *tBur* expression was driven in the epidermis using the *TH*-GAL4 and the *DDC*-GAL4 drivers in combination with *elav*-GAL80 to restrict expression to the epidermis. (B) Left panel: Abdominal pigmentation in 3h- (upper panel) and 48h-old (lower panel) males expressing *tBur* or *rk* RNAi transgenes under the control of pan neuronal drivers (*elav*-GAL4 *and nsyb*-GAL4). Right panel: Abdominal pigmentation in 3h- (upper panel) and 48h-old (lower panel) male flies expressing *tBur* or *rk* RNAi transgenes under the control of *rk*-GAL4 and restricted to non-neuronal cells using *elav*-Gal80. Genotypes are coded as described in Fig. 1B; boxes mark the first and third quartiles, thick lines mark the medians, and whiskers represent data range. Red dashed line indicates the median pigmentation level when *tBur* is expressed ubiquitously (*rk*>*tBur*). Results for each age were compared using a one-way ANOVA followed by Tukey HSD *post-hoc* analysis. Different letters indicate statistically significant differences (p< 0.001). NS: non-significant. n= 10 for each group.

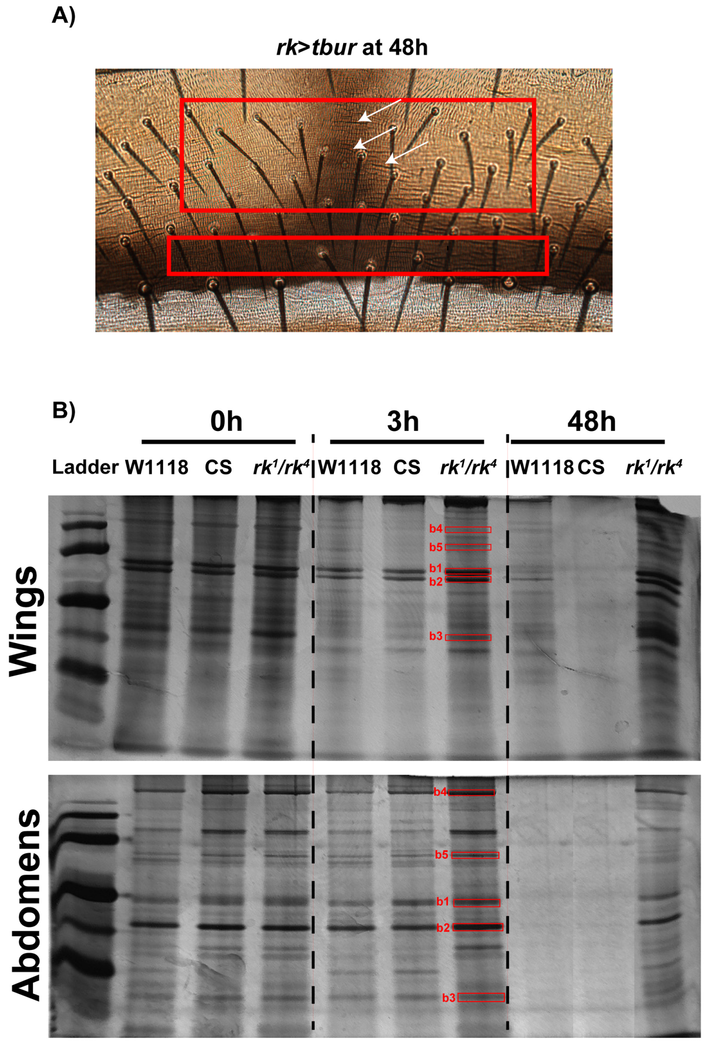

Figure S2. Details of methodology used to quantify abdominal cuticle pigmentation and sclerotization. (A) Picture of the fourth abdominal segment of a 48h-old *rk>tBur* female fly. To quantify melanization, we took two measurements (marked with red rectangles) in the central part of the segment (one in the upper part of the segment and one in the pigmented band at posterior edge of segment). This was the best approach in order to be able to compare the results at 3h vs. those of 48h. Indeed, the pigmentation difference between control and experimental groups at 3h post emergence was greatest in the pigmented band at posterior edge of segment (which darkens first), whereas at 48h this difference was greatest in the upper part of the segment. White arrows indicate microfolds that could only be seen in some experimental groups (see text). (B) Representative picture of silver-stained gel showing soluble cuticular proteins extracted from wings (upper) or abdominal epidermis (lower) of w^{1118}, CS, and rk^1/rk^4 flies aged 0h, 3h and 48h post-emergence. The 5 bands that were reliably seen in all gels and genotypes are outlined in red rectangles and were used to quantity the sclerotization state of the cuticle. The intensity of these bands was measured using ImageJ and expressed as a percentage of the intensity of the corresponding band obtained for w^{1118} control flies using the same protein extract for all experiment (see Methods). Note that the 5 bands used in wings *versus* abdomens are not necessarily the same. The intensity of the strongest band (band 2, b2) was used for the main figures.

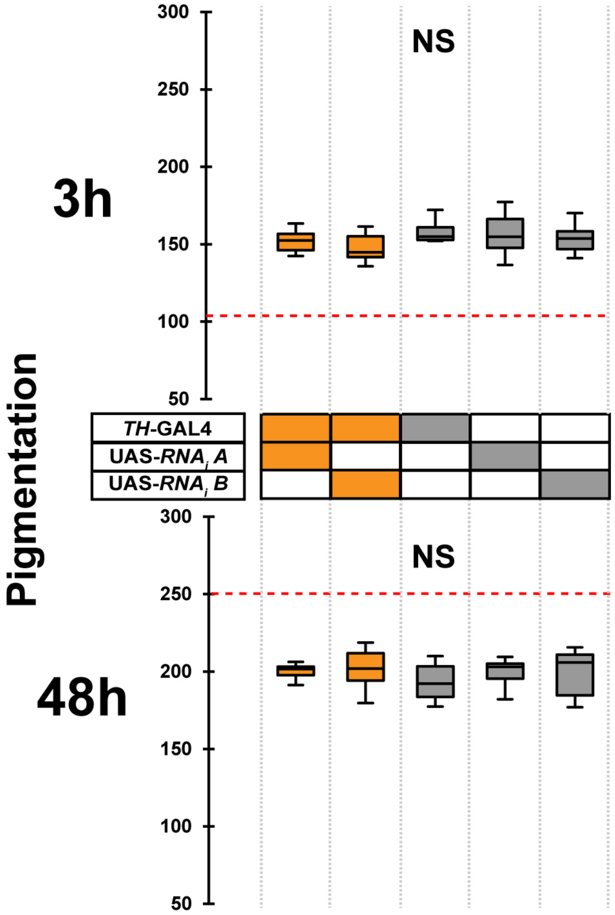

Figure S3. ***rk* knockdown in the epidermis does not induce pigmentation defects.** Abdominal pigmentation in 3h- (upper panel) and 48h-old (lower panel) females expressing *rk* RNAi transgenes under the control of *TH*-GAL4 driver. Genotypes are coded as described in Fig. 1B; boxes mark the first and third quartiles, thick lines mark the medians, and whiskers represent data range. Red dashed line indicates the median pigmentation level rk^1/rk^4 mutants. Results for each age were compared using a one-way ANOVA (p>0,41). NS: non-significant. n= 10 for each group.

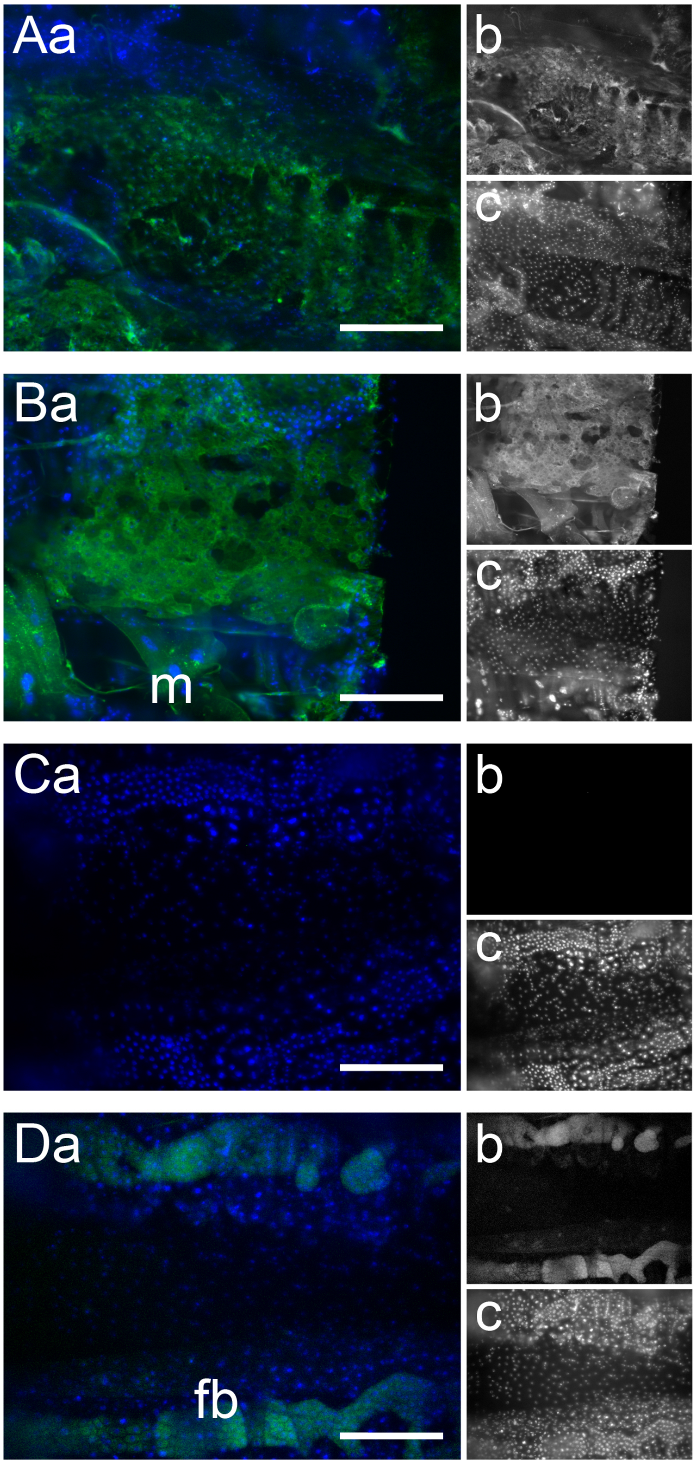

Figure S4. Epidermal expression of GAL4 drivers used in this study. GFP expression (green in panels [a] and shown alone in panels [b]) in single segment of dorsal abdominal epidermis of 3h-old female, driven by *TH*-GAL4 (A), *rk*-GAL4 (B), *dimm*-GAL4 (C), and *amon*-GAL4 (D). Preparations were counterstained with DAPI (blue in main panels [a] and shown alone in panels [c]) to visualize the nuclei. For these preparations overlying muscle was removed but remnants were sometimes present (e.g., m in panel Ba); fb (in Da) corresponds to fat body. Scale bar= 100 μm.

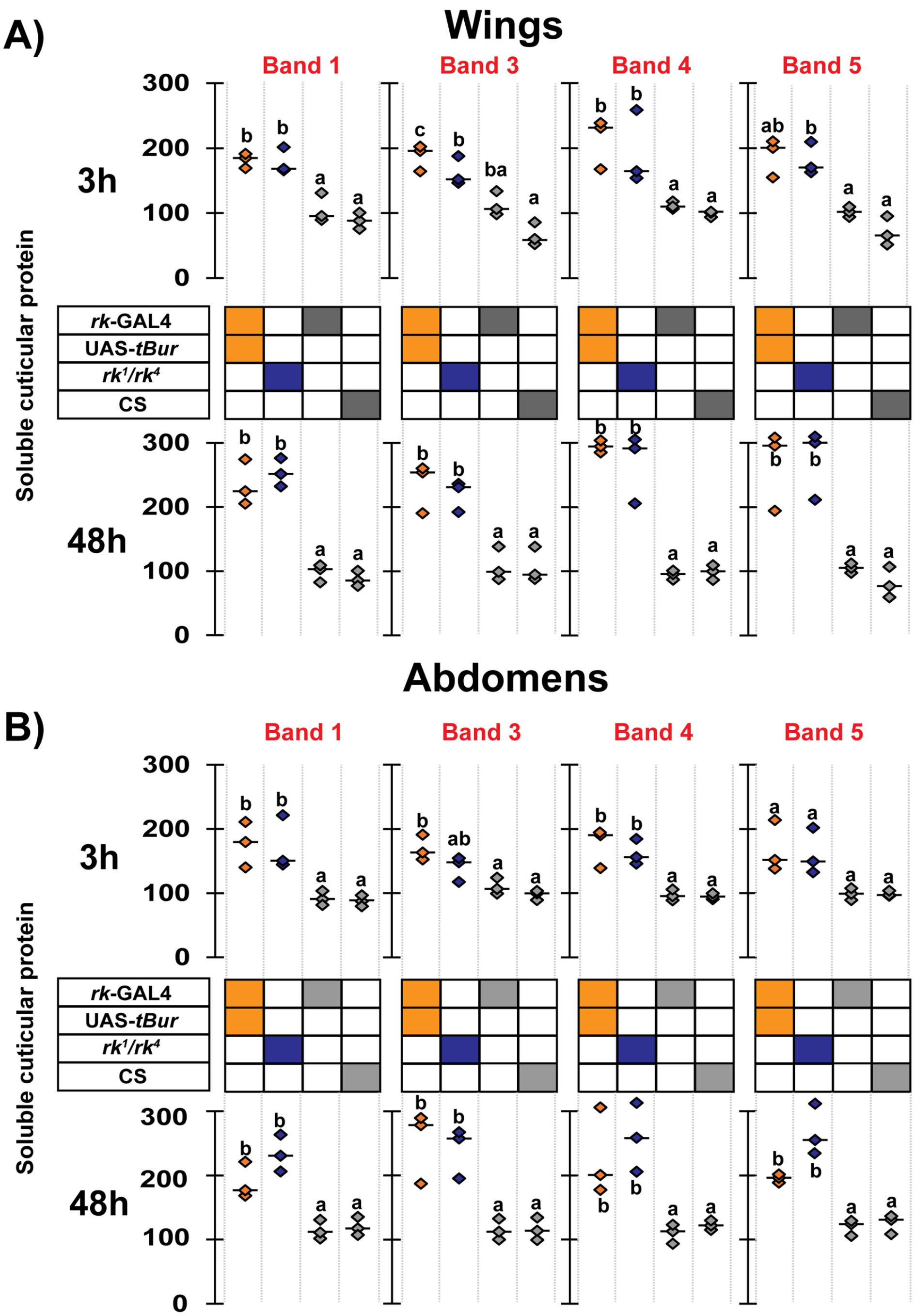

Figure S5. Sclerotization, quantified using other protein bands, in *rk* mutant females and in females expressing ubiquitous knockdown of RK function. Intensity of 4 other reference bands (see Additional file 1: Figure S2B) from 3 separate experiments in *rk*[1]/*rk*[4] mutant animals, in *rk*>*tBur* female flies, and in their respective controls. (A) Quantification of soluble cuticular proteins extracted from wings at 3h (upper panel) and 48h (lower panel) post emergence. (B) Quantification of soluble cuticular proteins extracted from abdomens at 3h (upper panel) and 48h (lower panel) post emergence. Short black lines indicate the median values. Genotypes are coded as described in Fig. 1B; different letters indicate statistically significant differences (one-way ANOVA followed by Tukey HSD, $p < 0.01$)

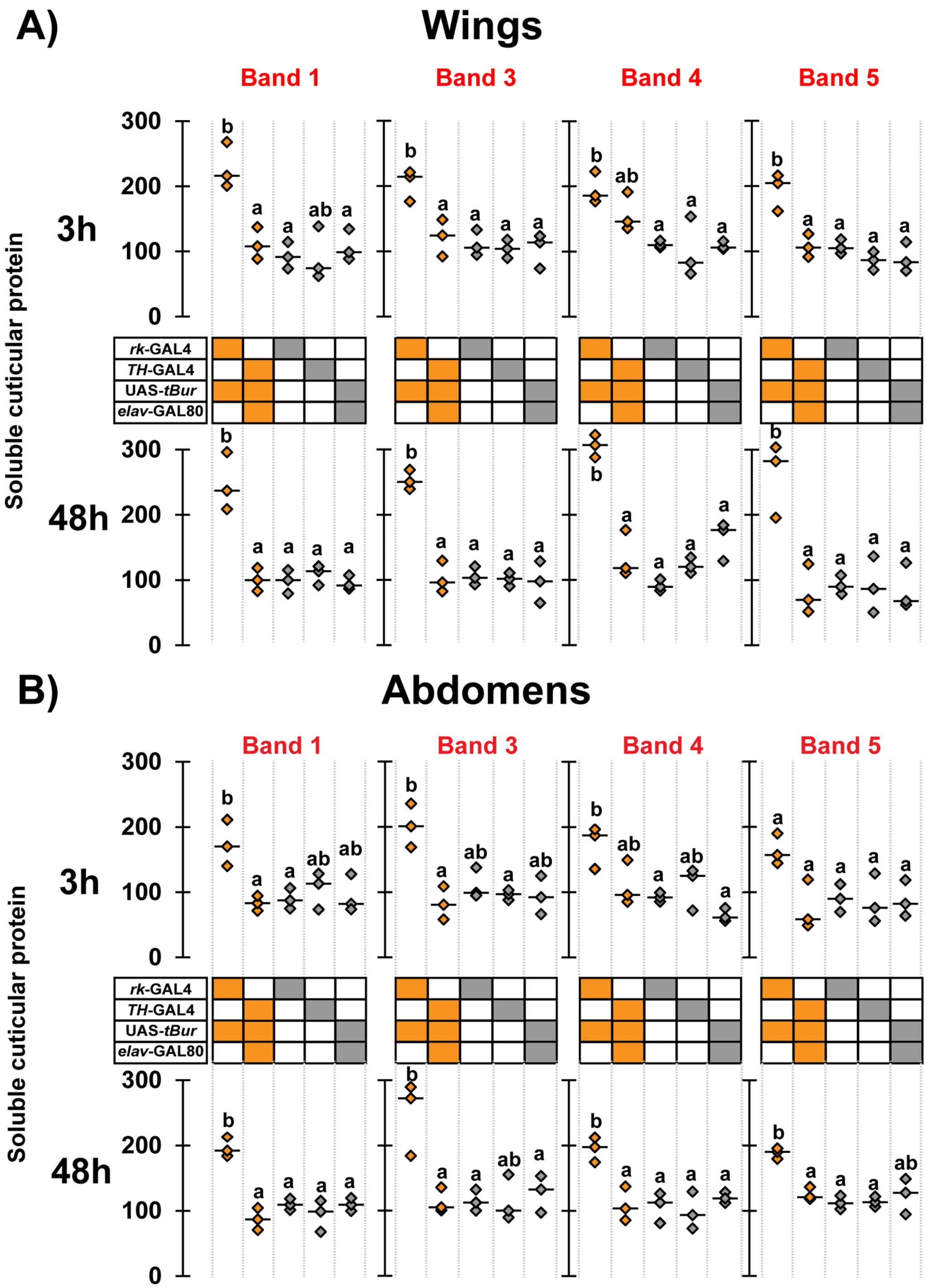

Figure S6. Sclerotization, quantified using other protein bands, showing that RK function is not required in the epidermis to regulate sclerotization. Intensity of 4 other reference bands (see Additional file 1: Figure S2) from 3 separate experiments in *TH>tBur*; *elav*-GAL80 and *rk>tBur* female flies and their respective control. (A) Quantification of soluble cuticular proteins extracted from wings 3h (upper panel) and 48h (lower panel) post emergence. (B) Quantification of soluble cuticular proteins extracted from abdominal epidermis at 3h (upper panel) and 48h post emergence (lower panel). Black lines indicate the median values. Genotypes are coded as described in Fig. 1B; different letters indicate statistically significant differences (one-way ANOVA followed by Tukey HSD, p< 0.01).

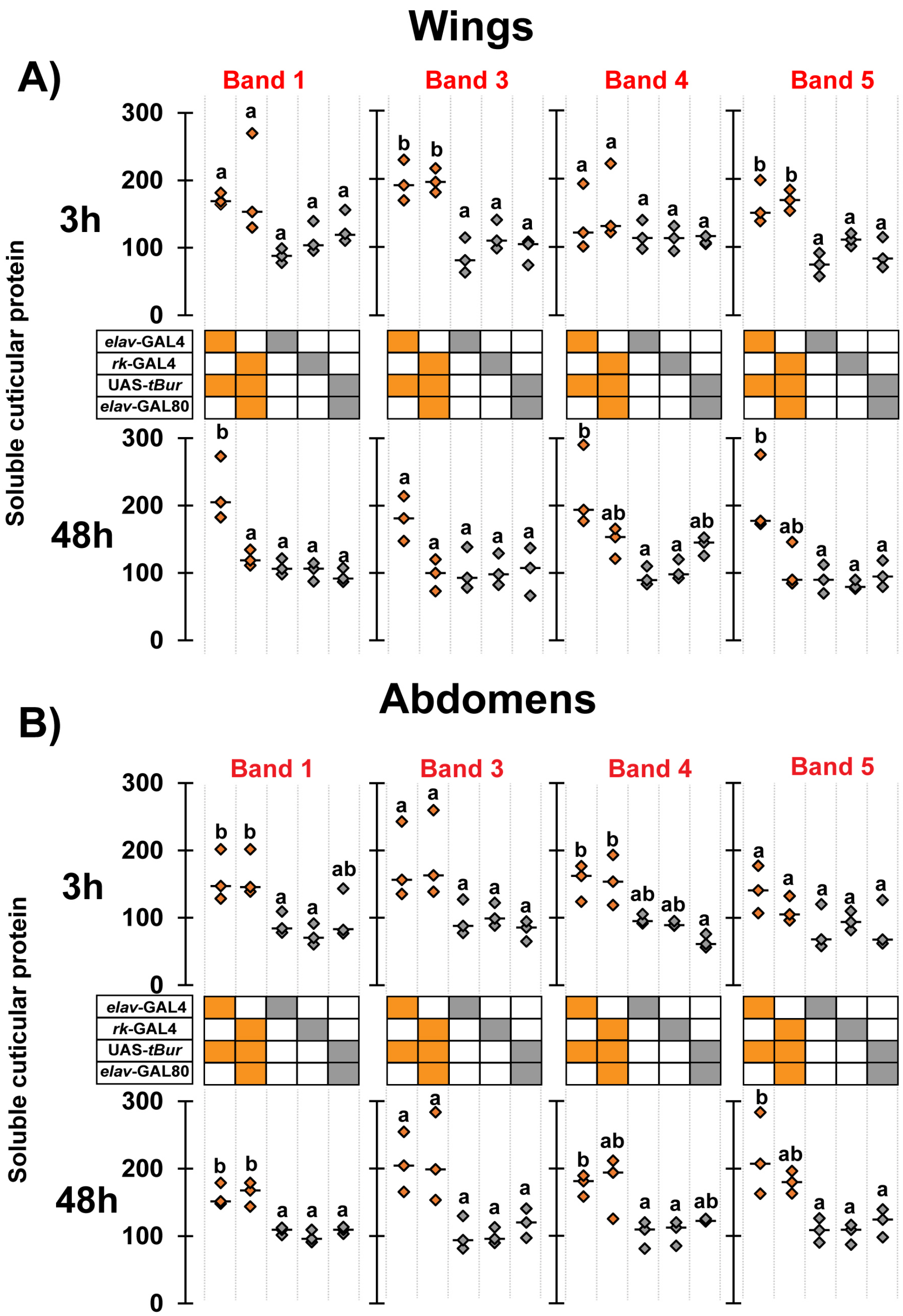

Figure S7. Sclerotization, quantified using other protein bands, show that RK function is necessary but not sufficient in the CNS to regulate sclerotization. Intensity measured using 4 other reference bands (see Additional file 1: Figure S2B) from 3 separate experiments in *rk>tBur*; *elav*-GAL80 and *elav>tBur* female flies and their respective control. (A) Quantification of soluble cuticular proteins extracted from wings at 3h (upper panel) and 48h (lower panel) post emergence. (B) Quantification of soluble cuticular proteins extracted from abdominal epidermis at 3h (upper panel) and 48h (lower panel) post emergence. Black lines indicate the median values. Genotypes are coded as described in Fig. 1B; different letters indicate statistically significant differences (one-way ANOVA followed by Tukey HSD, $p < 0.01$).

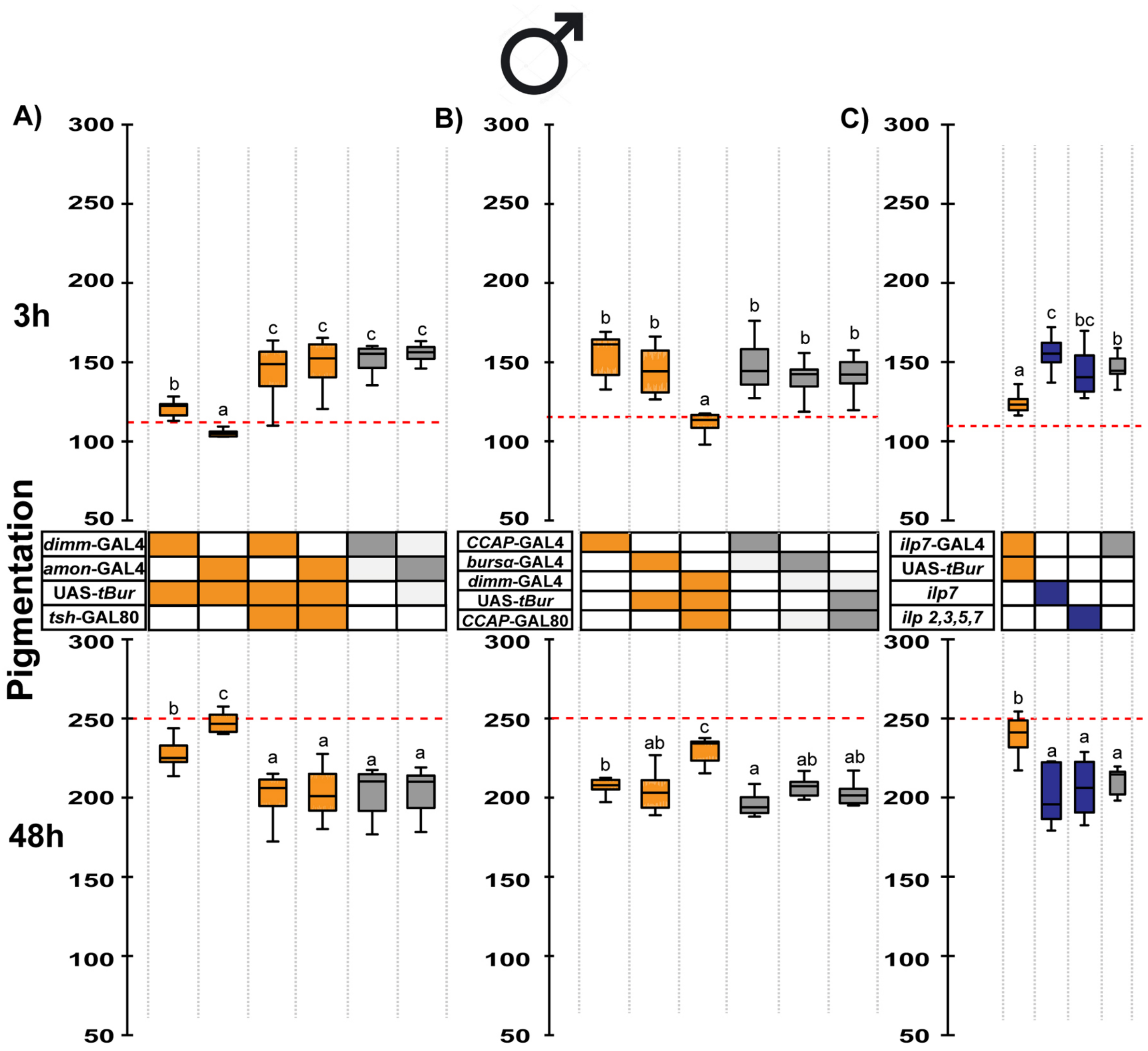

Figure S8. Abdominal pigmentation in males in which RK function was downregulated in peptidergic, CCAP, and ILP7 neurons. (A) Abdominal pigmentation in 3h- (upper panel) and 48h-old (lower panel) male flies expressing *tBur* under the control of peptidergic neuron drivers (*dimm*-GAL4 and *amon*-GAL4) and restricted to the VNS using *tsh*-GAL80. (B) Abdominal pigmentation in 3h- (upper panel) and 48h-old (lower panel) female flies expressing *tBur* in: bursicon neurons using CCAP-GAL4, and in peptidergic neurons using the *dimm*-GAL4 and restricted to non-CCAP neurons using CCAP-GAL80. (C) Abdominal pigmentation in 3h- (upper panel) and 48h-old (lower panel) male flies expressing *tBur* in ILP7 neurons using *ilp7*-GAL4; in *ilp7* null mutant flies and in flies (multiply) mutant for *ilp2*, *ilp3*, *ilp5*, and *ilp7*. Genotypes are coded as described in Fig. 1B; boxes mark the first and third quartiles, thick lines mark the medians, and whiskers represent data range. Red dashed line indicates the median pigmentation level when *tBur* is expressed ubiquitously (*rk*>*tBur*). Results were compared using a one-way ANOVA at each age followed by Tukey HSD *post-hoc* analysis. Different letters indicate statistically significant differences ($p < 0.001$). NS: non-significant. n= 10 for each group.

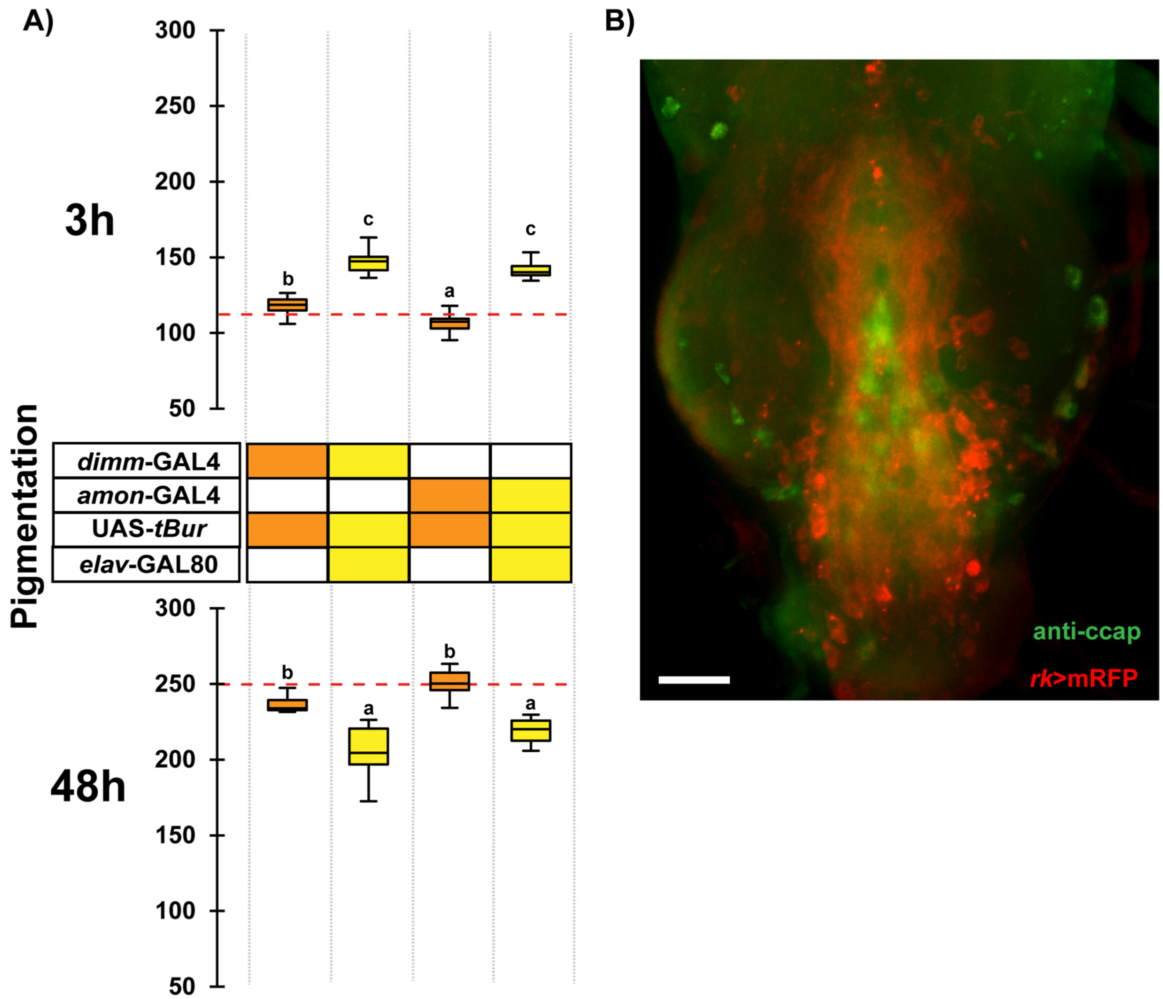

Figure S9. RK function is necessary in peptidergic neurons that are not CCAP immunopositive to regulate melanization. (A) Abdominal pigmentation in 3h- (upper panel) and 48h-old (lower panel) female flies expressing *tBur* in peptidergic cells using *dimm*-GAL4 and *amon*-GAL4 and restricted to non-neuronal tissue using *elav*-GAL80. Boxes indicate the first and third quartiles, thick central lines mark the medians, and whiskers represent data range. Red dashed lines indicate the median pigmentation level when *tBur* is expressed ubiquitously (*rk*>*tBur*). n=10 in each group. Results for each age were compared using a one-way ANOVA followed by a Tukey HSD *post-hoc* analysis. Different letters indicate statistically significant differences (*p*-values ≤0.001). Genotypes are coded as described in Fig. 1B except that orange and yellow were used to indicate that the GAL4 driver was used alone (orange) or in combination with *elav*-GAL80 (to restrict GAL4 expression to non-neuronal tissues). (B) Abdominal ganglion of a 1h old female in which a mCD8-tagged red fluorescent protein was expressed in all *rk* cells (*rk>mRFP*) and immunostained using an antibody against CCAP. Scale bar= 25 µm.

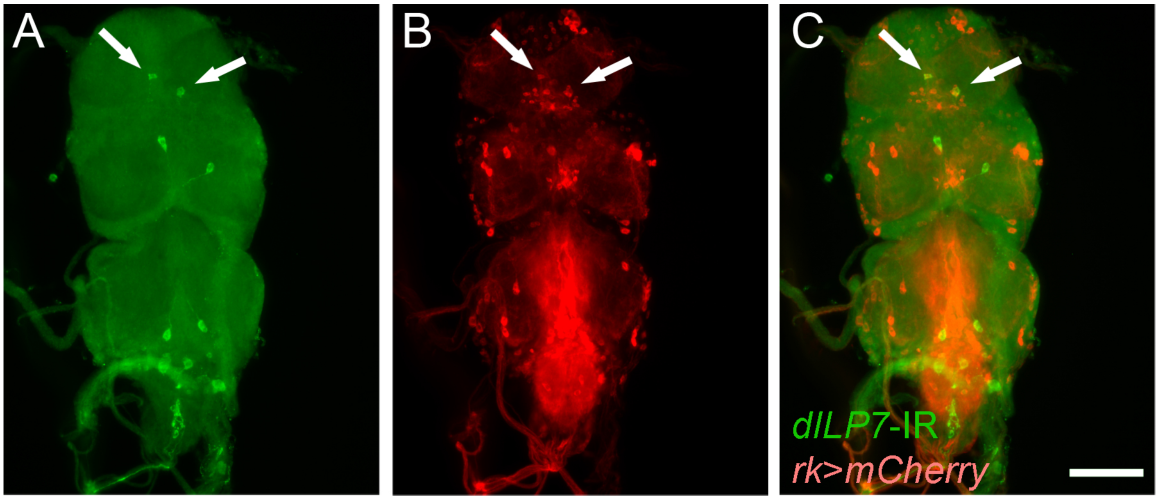

Figure S10. Some ILP7-immunopositive neurons express *rk*. Ventral nervous system of a 1h old female immunostained for ILP7 (A; in green) and expressing mCherry fluorescent reporter driven by *rk*-GAL4 driver (*rk>mCherry*; B) [27]. (C) Shows merged picture. Arrows point to a pair of dILP7-immunopositive neurons that express *rk* reporter. Scale bar in (C): 75 µm

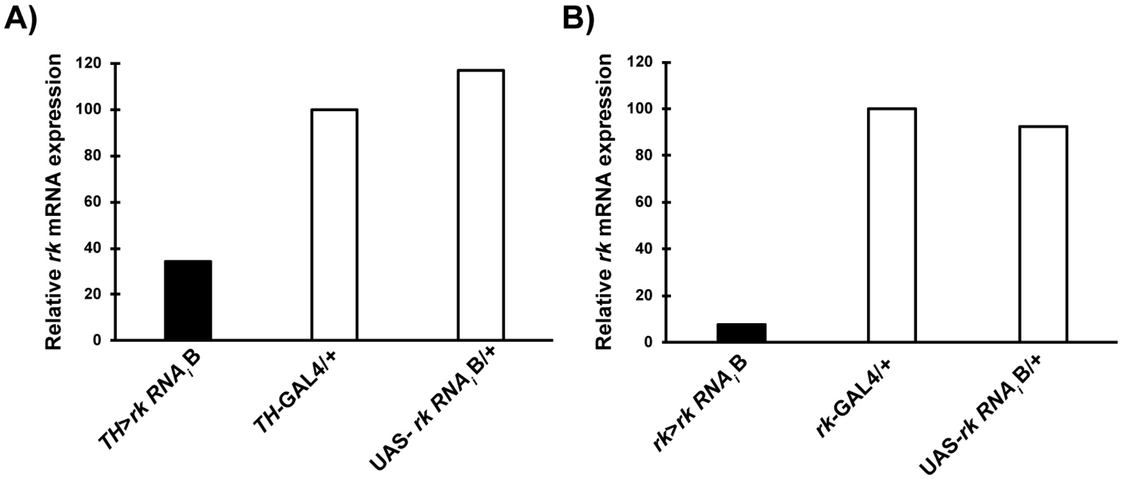

Figure S11. *rk* knockdown efficiency. Relative *rk* RNA expression in abdominal epidermis of 0-1h old flies for which *rk* RNAi was driven using *TH*-Gal4 (A) and *rk*-Gal4 (B) drivers. *rk* RNAi downregulated *rk* expression around 3 fold for *TH>rk* RNAi (A) and around 13 fold for *rk>rk* RNAi (B).
